# Molecular identification, biomass, and biochemical composition of the marine chlorophyte *Chlorella* sp. MF1 isolated from Suez Bay

**DOI:** 10.1186/s43141-020-00044-8

**Published:** 2020-07-09

**Authors:** Mostafa El-Sheekh, Mahmoud Abu-Faddan, Atef Abo-Shady, Mohamed Zein Alabedin Nassar, Wagdy Labib

**Affiliations:** 1 Botany Department, Faculty of Science, Tanta University, Tanta, Egypt; 2 Marine Environment Division, National Institute of Oceanography and Fisheries, Cairo, Egypt

**Keywords:** 18S rRNA, Growth, Biomass, Lipid, Fatty acids, Carbohydrate, protein, *Chlorella* sp

## Abstract

**Background:**

An Egyptian indigenous unicellular green microalga was isolated from the coastal water of Suez Bay (N 29.92°, E 32.473°), Red Sea, Egypt. The molecular analysis based on 18S rRNA sequence showed that the gene sequence for this strain was highly similar (100% identity and 98% query cover) to different *Chlorella* strains isolated from different habitats.

**Results:**

The observed morphological characters together with the molecular phylogeny assigned the isolated microalga as *Chlorella* sp. MF1 with accession number KX228798. This isolated strain was cultivated for estimation of its growth and biochemical composition. The mean specific growth rate (*μ*) was 0.273 day^−1^. Both the biomass productivity and the cellular lipid content increased by increasing salinity of the growth medium, recording a maximum of 6.53 g_DW_ l^−1^ and 20.17%, respectively, at salinity 40.4. Fourteen fatty acids were identified. The total saturated fatty acid percentage was 54.73% with stearic (C18:0), arachidic (C20:0), and palmitic acids (C16:0) as major components, while the total unsaturated fatty acid percentage was 45.27% with linoleic acid (C18:2c) and oleic acid (C18:1) as majors.

**Conclusion:**

This algal strain proved to be a potential newly introduced microalga as one of the most proper options available for microalgae-based biodiesel production. The proximate analysis showed the protein content at 39.85% and carbohydrate at 23.7%, indicating its accessibility to various purposes.

## Background

Microalgae are a ubiquitous group of fast-growing unicellular or simple multicellular microphototrophs. Environmentally, microalgae cultivation is considered a promising solution which mitigates global warming via sequestering the primary atmospheric greenhouse gas, CO_2_, by photosynthesis [1, 2]. Screening of local microalgae species with high nutritional value and potential for oil production is essential to achieve successful commercial large-scale cultures. The species of the genus *Chlorella* are considered as cryptic species that are morphologically similar but genetically distinct [3, 4]. The lack of obvious morphological taxonomic characteristics in addition to an exclusively asexual reproductive cycle by means of autospores makes it difficult to differentiate between the species of the genus *Chlorella* Beijerinck depending on the traditional taxonomy [5]. Sequences for 18S rRNA gene were used to identify several microalgal species and in particular to differentiate species of *Chlorella* [6, 7]. In recent years, microalgae achieve high potential as a feedstock for biofuel production due to their several advantages such as higher biomass productivity, lesser water demand, and no agricultural land requirement compared to other energy crops [1, 8]. Furthermore, microalgae production and accumulation of proteins, carbohydrates, lipids, and carotenoids are of particular importance when the microalgae are cultivated either to feed human and marine animals or to produce specific valuable substances, for instance [9]. All recent applications on microalgae are favored by high productivity and a precious chemical profile of the cultivated species. However, the chemical content of microalgae varies widely due to differences with culture density, age, changes in culture conditions, methods of measurement used, and the physiological state of the microalgae and to the growth phase of the culture [10, 11]. Continuous and semi-continuous cultures have gained attention not only for providing a constant source of microalgal biomass, but also for permitting precise manipulation of their biochemical composition [12]. The growth rate of most microalgae species and their biochemical composition, the protein, lipid, and carbohydrate contents, are influenced by alteration in the physiochemical parameters of the growth medium such as light intensity, pH, salinity, temperature, and nitrogen [13, 14]. The ability to constantly sense and rapidly adapt to environmental changes is paramount for microalgae to maintain cellular functions (homeostasis) [15]. Salinity is one of the most important factors affecting the growth of marine microalgae [16]. Generally, microalgae biomass comprises 40–60% proteins, 20–30% carbohydrates, and 10–20% lipids [17]. Lipids are a very attractive feedstock for biofuel production because of their high energy content and easy transforming to biodiesel [18]. The fatty acid composition of the algal lipids is a significant determinant of the biodiesel quality [19]. The most important properties of biofuel, such as cetane number and kinematic viscosity, are highly associated with the structural features of fatty acids [20, 21]. Recently, the lipid-free microalgal biomass plays an important role for economic biodiesel production, could be anaerobically digested for biomethane production [22], enhances the nutritional value of conventional food preparations [23], and can benefit terrestrial plant growth as a fertilizer [18]. The carbohydrate content of microalgae is mainly used as a feed source for livestock or in aquaculture for production of bivalve, crustaceans, and some fish species [24]. Also, carbohydrates can be used as a sugar source in an alcohol fermentation process for bioethanol production [25, 26]. Furthermore, microalgae are a very promising source for the synthesis of single cell protein or microbial protein that is a candidate to be sustainable future protein source to fill the deficit of the human need for protein [27].

This study aimed to isolate, identify, and assess growth and chemical composition of a marine chlorophyte, *Chlorella* sp. MF1, in continuous culture in the context of the possible use of microalgae as food species in mariculture and as feedstock for biofuel production.

## Methods

### Isolation and culturing of a microalga

A microalga was isolated from water samples collected in July 2013 from the coastal water at of Suez Bay (N 29.92°, E 32.473°), Egypt. It was morphologically identified under a microscope as *Chlorella* sp. Isolation and purification were performed by micropipette washing and streak plating techniques according to Phang and Chu [28] using BG11 medium.

### Molecular analysis

Cells of *Chlorella* sp. were harvested at the exponential growth phase by centrifugation. The algal pellet was washed three times with polysaccharide elimination buffer [29]. The DNA was extracted, to amplify the sequences of 18S ribosomal RNA gene, using the protocol of GeneJet Plant genomic DNA purification Kit (Thermo) # K0791. PCR amplification of the partial 18S ribosomal RNA genes was performed using the following primer set: the forward oligo (5′-CCT GGT TGA TCC TGC CAG-3′) and the reverse oligo (5′-A/TTG ATC CTT CT/CG CAG GTT CA-3′). Polymerase chain reaction (PCR) was carried out using Maxima Hot Start PCR Master Mix (Thermo) #K0221 using the recommended thermal cycling conditions outlined in Table 1. Evolutionary analyses were conducted in MEGA6 software [30] using the maximum likelihood method based on the Kimura 2-parameter model [31].

**Table 1:**
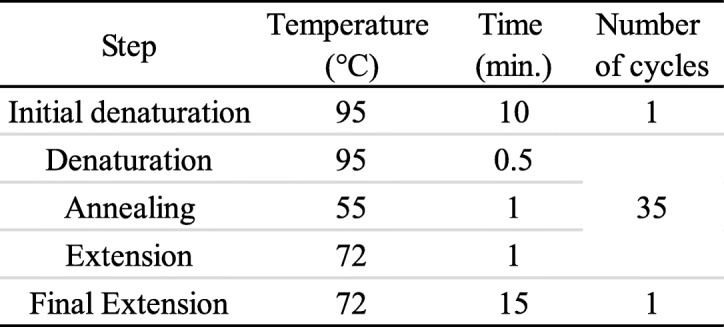
PCR conditions used in this study

### Culture conditions

The microalgal strain *Chlorella* sp. MF1. (Accession No: KX228798), isolated from Suez Bay, was grown in BG11 broth medium [32] using filtered autoclaved seawater (salinity 40.4 and pH of 7.5). The culture was incubated for 15 days at 29 ± 1 °C in sterile 1000 mL Erlenmeyer flasks containing 500 mL media with 10% inoculum. The culture was provided with light/dark conditions of 16:8 h and gently daily agitated.

### Microalgal growth determination

The growth of the culture was measured every 24 h using two different methods: determining cell densities (cells/ml) using a hemocytometer cell counting [33], and measuring the optical density (OD) at a wavelength of 750 nm as recommended by Griffiths et al. [34] by a UV/V spectrophotometer (JENWAY 6705, Staffordshire, UK). All measurements were done in triplicate. The mean specific growth rate (*μ*) in the exponential phase was calculated by averaging *μ*_1_ and *μ*_2_, using the following equation:

$$ \mu \left({\mathrm{day}}^{-1}\right)=\left[{\mu}_1+{\mu}_2\right]/2 $$

where *μ*_1_ is the slope of the regression line detected from the regression equation that relates time (*T*) to the natural logarithm of cell density in the exponential phase (ln CD ml^−1^) calculated as ln CD ml^−1^ = constant + *μ*_1_*T. (*μ*, day^−1^), *μ*_2_ as [ln*X*_2_ − ln*X*_1_]/ [*t*_2_ – *t*_1_], where *X*_2_ and *X*_1_ are the optical density (OD_750_) at *t*_2_ and *t*_1_ respectively. The generation (doubling) time (*g*) was expressed as *g* (day) = ln(2)/*μ*. The growth rate constant (*K*) expressed in terms of *g* as *K* (generations day^−1^) = 1/*g*. The maximum biomass (*M*) is calculated as follows: *M* (cell ml^−1^) = *M*_*t*_ − *M*_0_, where *M*_*t*_ is the number of cells in the stationary phase with the maximum cell number and *M*_0_ is the inoculum number of cells. The equation of *μ*_2_ followed Liang et al. [35], and the others Madigan et al. [36].

Two liters of algal culture were harvested at late the logarithmic phase and used for further experiments, and the algal biomass was recovered from the medium by centrifugation (4500 rpm, 10 min). Then, the obtained cell biomass was lyophilized and stored at − 20 °C [37] for the subsequent analysis of protein and carbohydrate contents.

### Effect of salinity on biomass production and lipid content

Five salinity treatments (0.42, 8.41, 17.7, 28.28, and 40.4), each with three replicates, were conducted by mixing the fresh tap water (FW) with the filtered seawater (SW) in different proportions, 100% FW, 75% FW + 25% SW, 50% FW + 50% SW, 25% FW + 75% SW, and finally 100% SW. The cultures were aerated using an air pump. Microalgae are harvested at the end of the logarithmic phase by centrifugation at 4500 rpm for 10 min, washed twice with distilled water, and dried in an oven at 80 °C for 4 h. The total biomass was then determined gravimetrically and expressed in g L^−1^. The total lipid for each treatment was extracted using the modified method of Bligh and Dyer [38] with chloroform–methanol solvent. The solvent was removed by evaporation at 40 °C after, and the lipid content was determined gravimetrically. The major fatty acid composition was determined for lipid extracted from salinity 40.4 treatment, using GC analysis, according to Radwan [39].

### Estimation of protein and carbohydrate contents

Total carbohydrate content was measured following the method developed by Dubois et al. [40] as adopted by Herbert et al. [41]. Glucose standard solution was used to obtain a calibration curve, which was employed to calculate the carbohydrate content of the samples. Total soluble protein was determined quantitatively applying the method described by Lowry et al. [42]. The extinction was measured at 760 nm against blank. Using a calibration curve, constructed with bovine serum albumin as a reference, the protein concentration was estimated.

### Statistical analysis

Results are presented as the mean ± standard deviation (SD) from three replicates. Differences between treatments for the different measured variables were tested by one-way variance (ANOVA), followed by Tukey’s (HSD) test when significant differences were found (*p* < 0.05) in XLSTAT Version 2014.5.03.

## Results

### Morphology

**Fig. 1:**
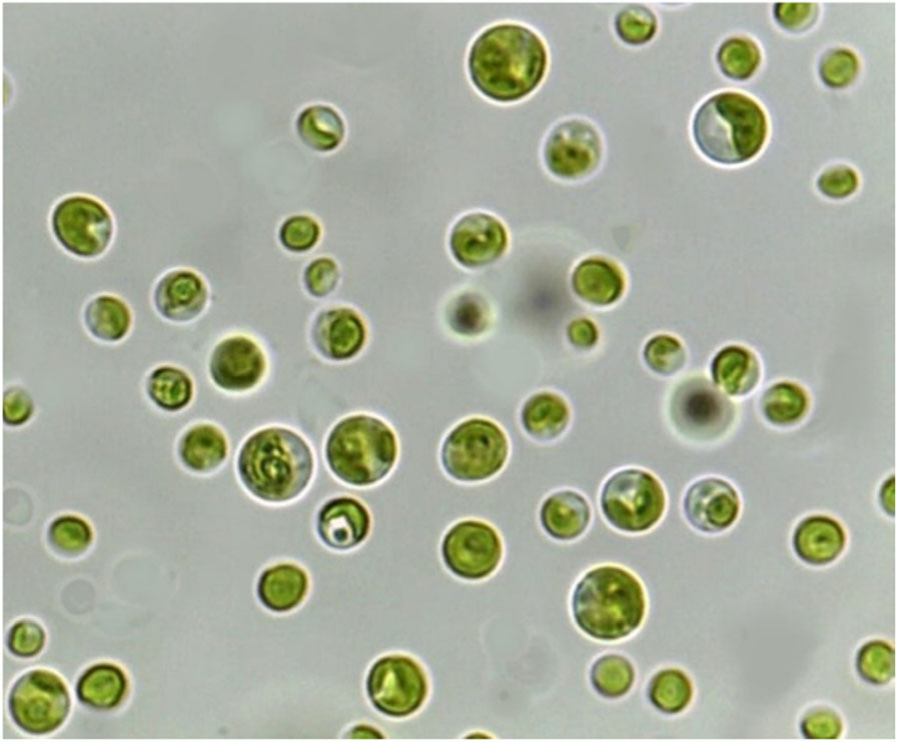
Photomicrograph of the young and mature vegetative *Chlorella* sp. MF1 cells under optic microscope (× 40)

The target microalgal isolate species examined with light microscopy (Fig. 1) was found to be green, unicellular, and spherical (coccoid) or subspherical. Chloroplast was parietal and cup-shaped with a single pyrenoid. In morphometric observations, cell diameter was 3–12 μm. Reproduction was by autosporulation, leading to two or more daughter cells; sexual reproduction is unknown. The following are the systematics of the species:

**Table:** 

Empire EUKARYOTA Kingdom PLANTAE Phylum CHLOROPHYTA Class TREBOUXIOPHYCEAE Order CHLORELLALES Family CHLORELLACEAE Genus CHLORELLA

### Phylogeny

**Fig. 2:**
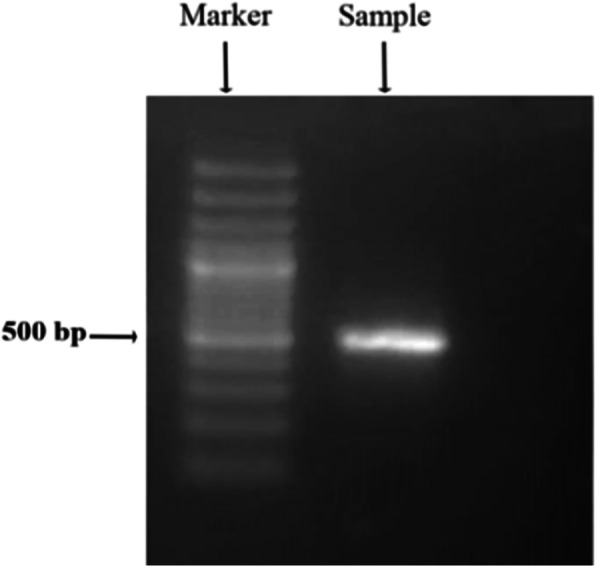
Agarose gel electrophoreses of the amplified 18S rRNA gene of *Chlorella* sp. MF1

In this study, identification of a Red Sea species of *Chlorella* was carried out using molecular and phylogenetic analyses of 18S rRNA nuclear gene. The genomic DNA of *Chlorella* sp. was extracted and the produced amplicons were electrophoresed in the agarose gel. Approximately 535 bp of amplified locus of 18S rRNA was observed (Fig. 2). All amplified fragments were sequenced and analyzed against NCBI database. Here, the molecular studies based on 18S rRNA allowed us to identify the isolated strain as *Chlorella* sp. MF1 with accession number KX228798. The gene sequences for the strain were highly similar (100% identity and 98% query cover) to different *Chlorella* strains isolated from different habitats. Furthermore, It was clearly observed that the sequences have a high percentage similarity which was approximately 100% with *Chlorella* sp. CBI small subunit ribosomal RNA gene, partial sequence (Accession No: MH125171) isolated from Indonesia.

**Fig. 3:**
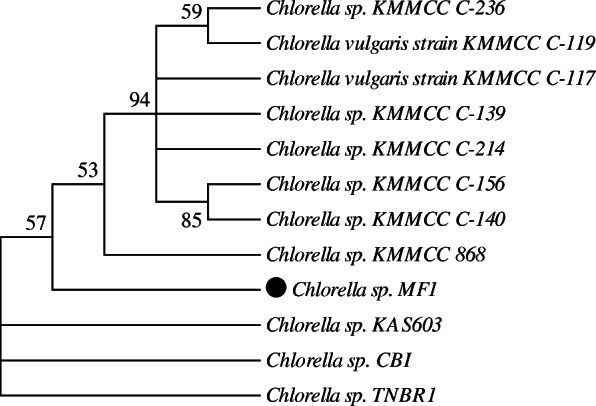
Maximum likelihood (ML) phylogenetic tree of *Chlorella* sp. MF1 based on partial sequencing of 18S rRNA. The cutoff value adopted is 50%. Bootstrap values were calculated from 1000 permutations. The formation of parallel branches is due to the similarity of sequences

The phylogenetic analyses (Fig. 3) resulted in a topology presenting unrooted tree that illustrates the relatedness of the leaf nodes and reflects the branching order without making assumptions about common ancestry [43]. The phylogenetic tree showed that the sequence of our strain was closely clustered with that of *Chlorella* sp. KAS603 and *Chlorella* sp. CBI (Fig. 3).

### Biomass productivity

**Fig. 4:**
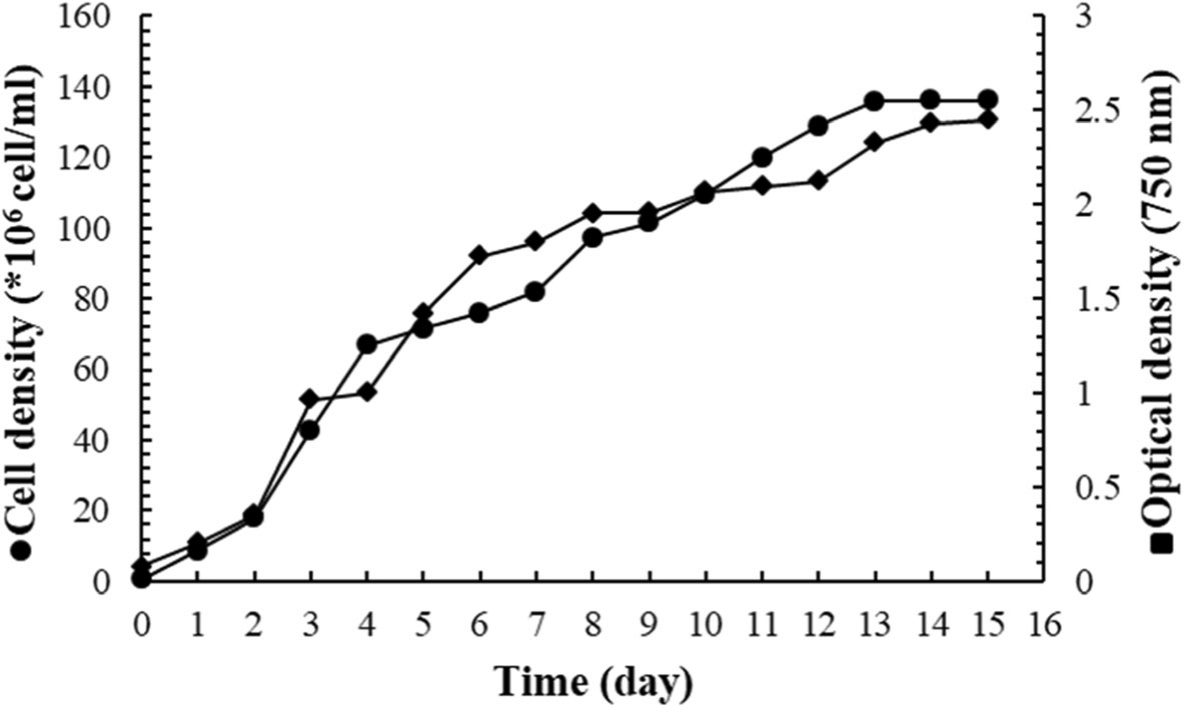
*Chlorella* sp. MF1 growth curve in BG11 medium

**Fig. 5:**
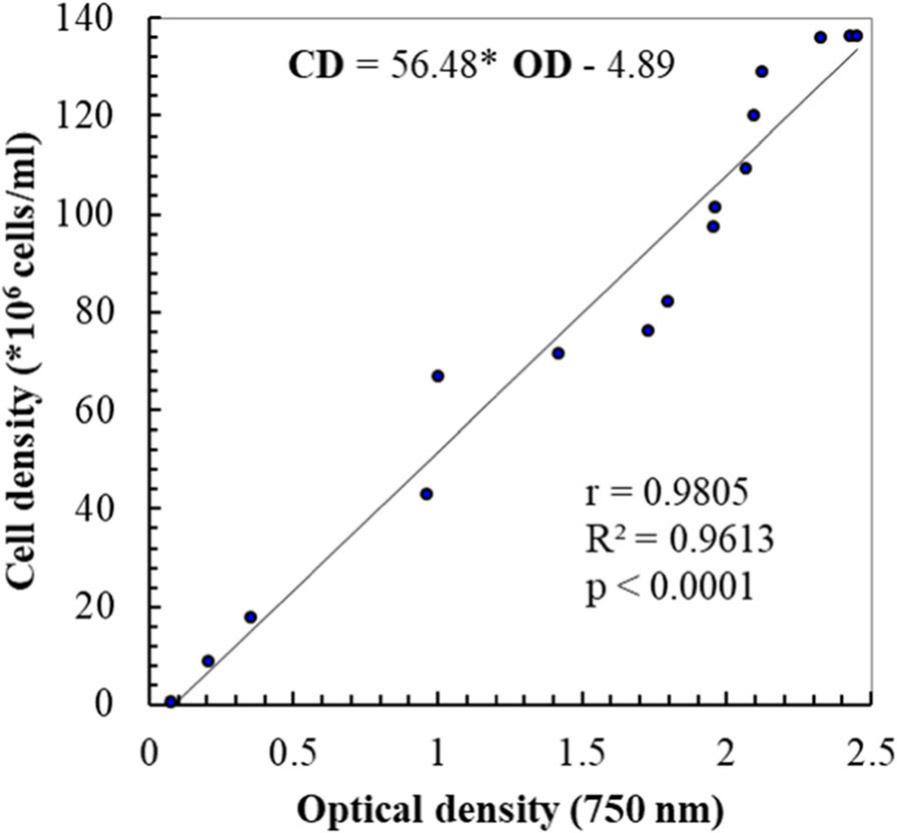
Relation between cell density and optical density of *Chlorella* sp. MF1

*Chlorella* sp. MF1 was cultivated for 2 weeks at salinity 40.4. The growth curve (Fig. 4) was determined after 1 day from incubation, and the growth reached exponential phase at the day 15, almost similar to the duration period of *Chlorella marina* reported by Muthukumar et al. [37]. The results indicate a significant linear relationship of the optical density (OD) at 750 nm with cells density (CD, cells * 10^7^/mL) as CD = 56.48* OD − 4.89 (Fig. 5); the regression equation relating time (*T*) to natural logarithm of cell density (ln CD/ml) expressed as ln CD/ml = 15.80757 + 0.2844*T; the slope of the regression line corresponds to specific growth rate calculated as *μ*_1_ = 0.284 day^−1^, while the specific growth rate based on cell density (*μ*_2_) was at 0.261 day^−1^. Also, the average of *μ*_1_ and *μ*_2_ resulted in the mean specific growth rate (*μ*) equal to 0.273 ± 0.01 day^−1^. The resulted generation time (*g*), the mean time required to double the population, was 2.54 days. Consequently, the growth rate constant (*K*), the number of generations per unit time, was at 0.393 generations/day, and the maximum biomass (*M*) was 135.65 × 10^6^ cells/ml (Table 2).

**Table 2:**
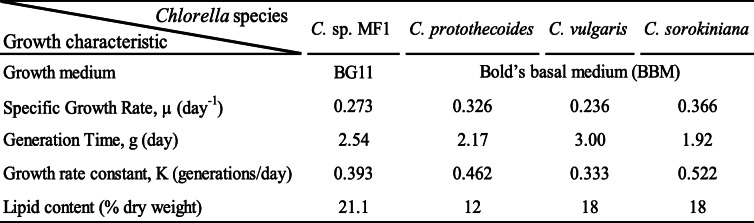
Growth characteristics of *Chlorella* sp. MF1 (this study) and other species of *Chlorella* based on Rosenberg et al. [44]

### Effect of salinity on growth rate and lipid content

**Fig. 6:**
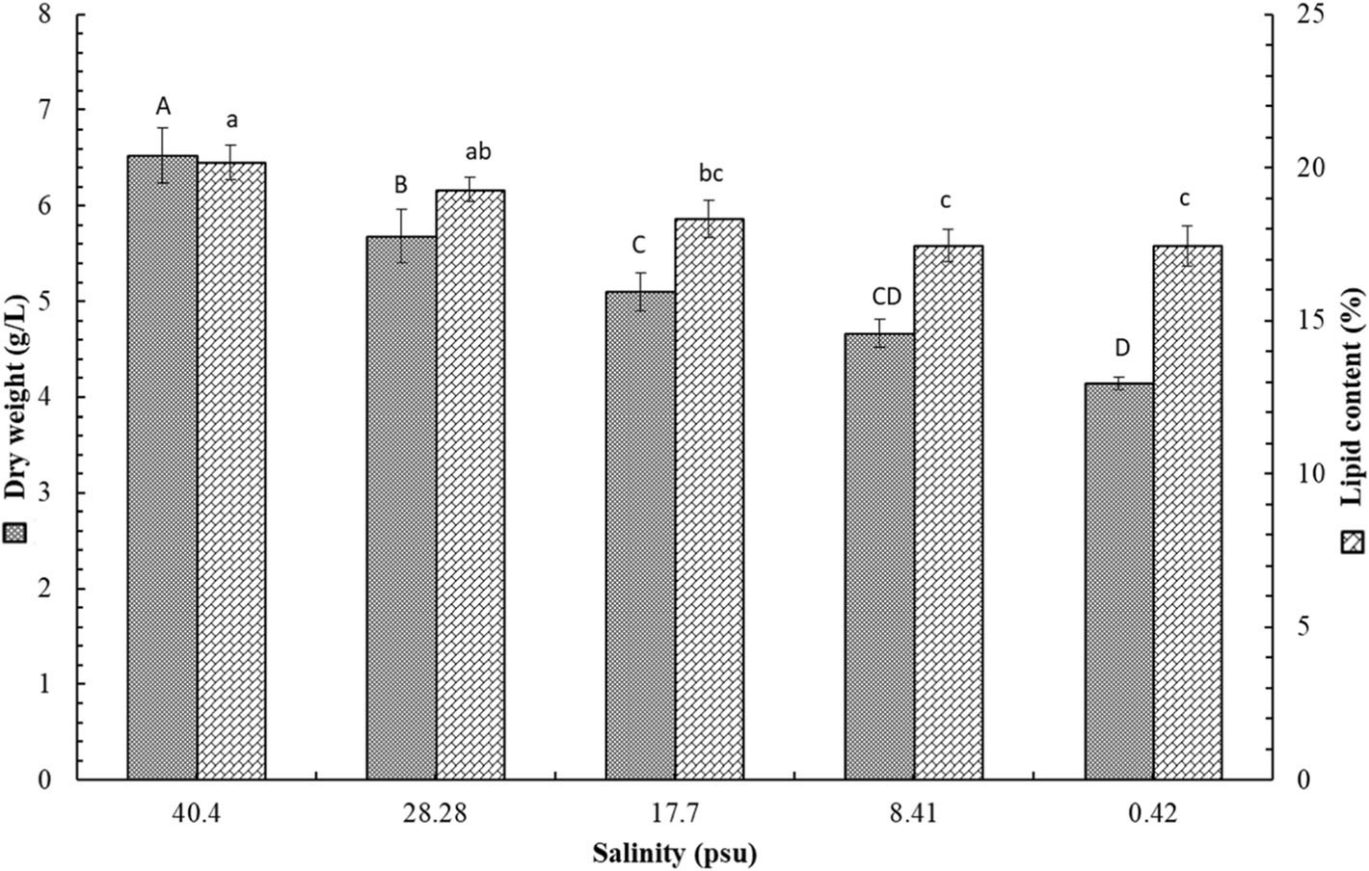
Biomass productivity (g l^−1^) and oil content (% oil/g of dry algal biomass) of *Chlorella* sp. MF1 under different salinity treatments. Different letters indicate statistically significant differences between the treatments using Tukey’s test

The results indicated that the biomass productivity ranged from a minimum of 4.147 ± 0.065 g_DW_ l^−1^ at salinity 0.42 (100% distilled freshwater) to a maximum of 6.53 ± 0.286 g_DW_ l^−1^ at salinity 40.4 (100% filtered seawater) with highly significant differences between salinity treatments (*p* < 0.05 by ANOVA) (Fig. 6).

The extracted greenish oil content (% oil/g of dry algal biomass) from the harvested algae ranged from a minimum of 17.447 ± 0.667% at salinity 0.42 to a maximum of 20.17 ± 0.574 at salinity 40.4 with significant differences between salinity treatments (*p* < 0.05 by ANOVA) (Fig. 6).

### Fatty acid components

The results showed that the fatty acid content as a percentage of total lipids (FAC/TL) was 14.13% (Table 3). The fatty acid content is mainly composed of a mixture of saturated (SFAC) and unsaturated (UFAC) fatty acids as 54.73% and 45.27% of total fatty acids, respectively. The major saturated fatty acids were stearic (C18:0), arachidic (C20:0), and palmitic acids (C16:0) while the major unsaturated were linoleic acid (C18:2c) and oleic acid (C18:1) (Table 4).

**Table 3:**
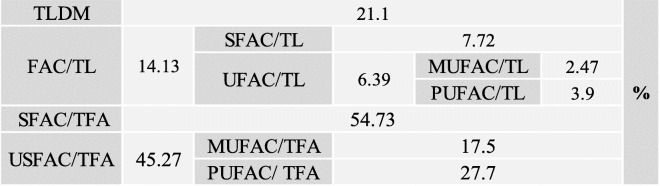
Total lipid content as percentage of dry mass (TLDM), saturated fatty acid content as percentage of total lipids (SFAC/TL) and total fatty acids (SFAC/TFA) and monounsaturated and polyunsaturated fatty acid content as percentages of total fatty acids (MUFAC/TFA and PUFAC/TFA, respectively) and of total lipids (MUFAC/TL and PUFAC/TL, respectively) and total fatty acid contents in the microalgae dry biomass

**Table 4:**
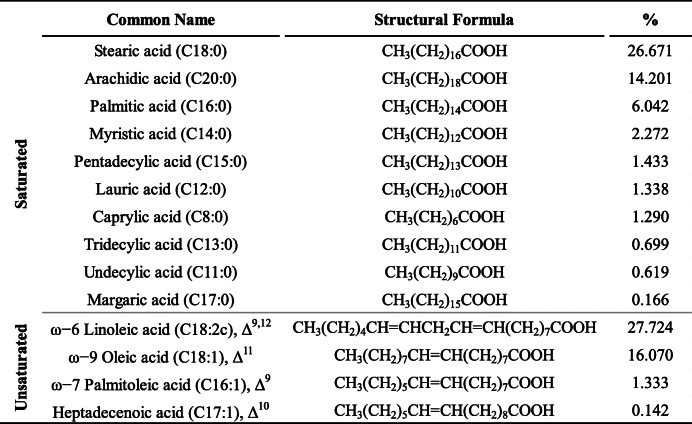
Fatty acid composition (% of total fatty acids) of *Chlorella* sp. MF1

### Protein and carbohydrate contents

**Fig. 7:**
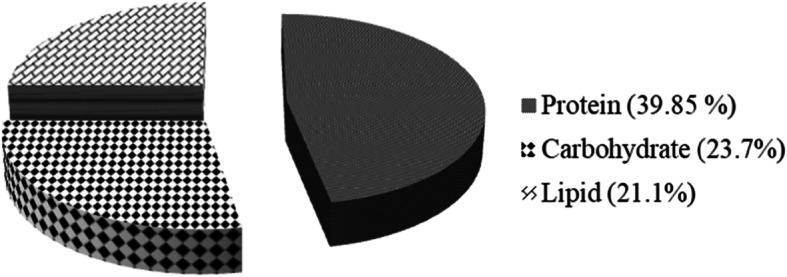
The biochemical composition of *Chlorella* sp. MF.1 (as percentage of dry weight)

The estimated protein content at the salinity 40.4 was 39.85% of dry weight, while the carbohydrate content was 23.7% (Fig. 7).

## Discussion

The microscopic observations of this work are in agreement with those recorded for the genus *Chlorella* Beijerinck [4, 45]. The phylogenetic analyses resulted in a topology presenting unrooted tree that illustrates the relatedness of the leaf nodes and reflects the branching order without making assumptions about common ancestry [43]. The rooted trees are ideally preferable, but almost every phylogenetic reconstruction algorithm provides an unrooted tree [46]. A polytomy or multifurcation is observed where there is no branching order consistent with 50% or more of the replicate trees. This reflects that bootstrap replications fail to support a branch in less than 50% of resamplings [47]. Also, polytomies reflect poorly resolved or conflicting relationships [48]. Future analyses of this data by other methods of phylogenetic inference may interpret some features of this tree. On the other hand, Basic Local Alignment Search Tool of the *National Center for Biotechnology Information* (NCBI BLAST) of *Chlorella* sp. MF1 sequence has shown close relationships (100% identity and 98% query cover) between highly dissimilar morphologies such as *Dicloster acuatus* strain Xmm25W2 and *Dictyosphaerium* sp. YN12-4, demonstrating that evolution of vegetative morphology can be rapid [49].

The growth of *Chlorella* sp. MF1 reached exponential phase at the day 15, almost similar to the duration period of *Chlorella marina* reported by Muthukumar et al. [37]. The resulted generation time (*g*), the mean time required to double the population, was 2.54 days. Consequently, the growth rate constant (*K*), the number of generations per unit time, was at 0.393 generations/day; and the maximum biomass (*M*) was 135.65 × 10^6^ cells/ml. This agrees to some extent with the results reported by Rosenberg et al. [44] for different *Chlorella* spp. The maximum biomass production is similar to that recorded for *Chlorella vulgaris* by Montoya et al. [50] using nitrogen-limited Bold basal medium provided with 4% CO_2_-enriched air, also by El-Mohsnawy et al. [51] under limited nitrogen and mixotrophic condition. The results indicated that the mean growth rate increased with increasing salinity in agreement with El-Sheekh et al. [8] who concluded that growing *Chlorella vulgaris*. under salt supplemented Kuhl medium (10 g/l NaCl) produces high biomass and oil for biodiesel production. However, Battah et al. [52] interpreted the growth inhibition of *Chlorella vulgaris* in response to salinity increase as a result of shifting the metabolites to the synthesis of osmoregulant compounds rather than synthesis of cellular constituents. The positive relation observed between the biomass productivity and salinity might be related to the excess of nutrients associated with the filtered seawater. Seawater contains more dissolved ions than all types of freshwater [53]. Generally, the higher seawater percentage in the growth medium, i.e, the higher salinity, leads to more nutrient loading in the medium and thereby increases biomass productivity. The role of salinity as an important stressor has been intensively investigated with different experimental techniques to spur lipid anabolism for biodiesel production [52, 54]. The extracted greenish oil content (% oil/g of dry algal biomass) from the harvested algae ranged from a minimum of 17.447% at salinity 0.42 to a maximum of 20.17 at salinity 40.4 with significant differences between salinity treatments, almost similar to that reported by Rosenberg et al. [44] for *Chlorella vulgaris* and *Chlorella sorokiniana*. Also, the results reinforce the conclusion that increasing salinity within a range that is specific for each species leads to increased cell lipid content as in *Navicula* sp. [55], *Dunaliella tertiolecta* [56], *Nannochloropsis oculata* [57], and *Chlorella vulgaris* [52].

The results showed that the fatty acid content as a percentage of total lipids (FAC/TL) was 14.13, close to the percentage of 15.4%, recorded for *Chlorella vulgaris* by Ohse et al. [58]. The high saturated fatty acid content (54.73% of TFA) when compared with unsaturated fatty acids (45.27% of TFA) provides the eligibility as a biodiesel feedstock. Production of biodiesel containing high saturated fatty acids is less susceptible to oxidation and rancidity [59]. These obtained results favor the marine microalga *Chlorella* sp. MF1 to be used as feedstock for biodiesel production and also as food and feed additives. Battah et al. [52] also recorded quasi-symmetric percentages, 53.99 and 46.01% respectively, for *Chlorella vulgaris* cultured in Bold basal medium containing 0.45 mM NaCl. The salt stress on the photosynthetic organisms, such as *Chlorella* sp. MF1, leads to an increase in desaturation of the fatty acids of membrane lipids [60, 61]. This increase reinforces the salt tolerance via increasing the plasma membrane viscosity and fluidity, increasing the turgor pressure of the cell and thus preventing the outflux of water from the cells as a mechanism of adaptation [62]. The major saturated fatty acids were stearic (C18:0), arachidic (C20:0), and palmitic acids (C16:0) while the major unsaturated were linoleic acid (C18:2c) and oleic acid (C18:1) (Table 4). These results almost agree with Battah et al. [52] who reported the linoleic acid as the major unsaturated, and palmitic the major saturated. Furthermore, palmitic, stearic, oleic, and linoleic acids were recognized as the most common fatty acids contained in biodiesel [19].

The estimated protein content at the salinity 40.4 was 39.85% of dry weight, lies in the range of 33–46%, for *Chlorella vulgaris* cultured under different light regimes [63]. The carbohydrate content was 23.7%, close to that recorded by Kim et al. [64] as 22.4% for *Chlorella vulgaris* cultured in Bold basal medium (BBM), but under nitrogen limitation conditions. Also, Laurens et al. [65] reported the total carbohydrate content in microalgae of about 22% dry weight (DW) and protein content about 44% DW.

## Conclusion

In conclusion, cultivation of *Chlorella* sp. MF1 is a new addition to the other species offering advantage as a vital marine source of biofuel production, human consumption, etc., but, further experiments are still needed. The high biomass productivity of such a marine microalgal species and the high contents of protein and fatty acids that have numerous health benefits indicate the accessibility to the previous purposes. Furthermore, it could be cultivated in seawater-enriched medium to save freshwater resources.

## Data Availability

All the data required for the processing of the conclusions are presented in the “Results” section.

## Abbreviations

PCR: Polymerase chain reaction
OD: Optical density
CD: Cell density
SFAC: Saturated fatty acids
UFAC: Unsaturated fatty acids
NCBI BLAST: Basic Local Alignment Search Tool of the *National Center for Biotechnology Information*
DM: Dry mass
TL: Total lipids
